# Characterizations of Plasticized Polymeric Film Coatings for Preparing Multiple-Unit Floating Drug Delivery Systems (muFDDSs) with Controlled-Release Characteristics

**DOI:** 10.1371/journal.pone.0100321

**Published:** 2014-06-26

**Authors:** Sheng-Feng Hung, Chien-Ming Hsieh, Ying-Chen Chen, Yu-Chun Wang, Hsiu-O Ho, Ming-Thau Sheu

**Affiliations:** 1 School of Pharmacy, College of Pharmacy, Taipei Medical University, Taipei, Taiwan; 2 Clinical Research Center and Traditional Herbal Medicine Research Center, Taipei Medical University Hospital, Taipei, Taiwan; 3 Department of Cosmetic Science, Providence University, Taiwan Boulevard, Shalu, Taichung, Taiwan, ROC; Taipei Medical University, Taiwan

## Abstract

Effervescent multiple-unit floating drug delivery systems (muFDDSs) consisting of drug (lorsartan)- and effervescent (sodium bicarbonate)-containing pellets were characterized in this study. The mechanical properties (stress and strain at rupture, Young’s modulus, and toughness) of these plasticized polymeric films of acrylic (Eudragit RS, RL, and NE) and cellulosic materials (ethyl cellulose (EC), and Surelease) were examined by a dynamic mechanical analyzer. Results demonstrated that polymeric films prepared from Surelease and EC were brittle with less elongation compared to acrylic films. Eudragit NE films were very flexible in both the dry and wet states. Because plasticizer leached from polymeric films during exposure to the aqueous medium, plasticization of wet Eudragit RS and RL films with 15% triethyl citrate (TEC) or diethyl phthalate (DEP) resulted in less elongation. DEP might be the plasticizer of choice among the plasticizers examined in this study for Eudragit RL to provide muFDDSs with a short time for all pellets to float (TPF) and a longer period of floating. Eudragit RL and RS at a 1∶1 ratio plasticized with 15% DEP were optimally selected as the coating membrane for the floating system. Although the release of losartan from the pellets was still too fast as a result of losartan being freely soluble in water, muFDDSs coated with Eudragit RL and RS at a 1∶1 ratio might have potential use for the sustained release of water-insoluble or the un-ionized form of drugs from gastroretentive drug delivery systems.

## Introduction

Based on recently published literature and patents applied for, gastroretentive drug delivery systems (DDSs, GRDDSs) that are retained in the stomach for a prolonged and predictable period of time are one of the advanced approaches for novel drug-delivery systems [1], [2]. GRDDSs are particularly appropriate for drugs with a narrow absorption window [3]–[5], drugs that act locally in a part of the gastrointestinal (GI) tract (GIT) such as antibiotic administration for *Helicobacter pylori* eradication to treat peptic ulcers [6]–[8], drugs which are unstable in intestinal fluids [3], [9], [10], and drugs that exhibit poor solubility in the intestinal tract [11], . The development of various approaches for GRDDSs, including low-density systems/floating systems, high-density systems/non-floating systems, mucoadhesive or bioadhesive systems, expansion systems, magnetic systems, supraporous hydrogels, and raft-forming systems, was reviewed [13], [14]. Low-density systems or floating DDSs (FDDSs) are further divided into non-effervescent and effervescent systems based on the mechanism of buoyancy. If their bulk density is lower than that of gastric fluid, and they thus remain buoyant in the stomach for a prolonged period. From formulation and technological points of view, FDDSs are considerably easy and a logical approach for developing GRDDSs.

Upon contact of an effervescent FDDS with gastric fluid, the fluid penetrates its outer layer (or membrane) and reacts with the effervescent components (e.g., sodium bicarbonate alone or combined with citric acid or tartaric acid). Carbon dioxide (CO_2_) is liberated causing the FDDS to float in the stomach due to its buoyancy effect and lower bulk density. Previously reported FDDSs were prepared as single-unit systems, such as tablets and capsules [15]. Nevertheless, the disadvantage of single-unit systems is the inter?/intra-subject variability of the GI transit time due to its all-or-nothing emptying processes [6], [16]–[19], which raises the possibility of dose dumping [20]. Hence, the concept of multiple-unit dosage forms, such as granules, pellets, and mini-tablets, was developed. Various effervescent multiple-unit FDDSs (muFDDSs) were reported to prolong gastric residence times and increase the overall bioavailability of the dosage form. Those systems demonstrated that using Eudragit RL alone or a combination of Eudragit RL and RS as the polymeric layer could cause floating for desirable periods (in the stomach for about 5 h *in vivo*) and possessed controlled-release properties [21]. The development of effervescent muFDDSs is a promising area of pharmaceutical research for controlled release in the stomach. As flexible dose adjustment and reducing subject variatons are expected to achieve via the dosage form.

An ideal membrane for effervescent muFDDSs should allow water to permeate at a fast enough rate in order to immediately activate the effervescent reaction, thus preventing the individual unit from transiting to the small intestine [22]. But hydrated films should also be impermeable to the generated CO_2_ to maintain floatation and remain sufficiently flexible to withstand the pressure of CO_2_ to avoid rupture [23]. Therefore, with an optimal permeability for water, ideal coating polymeric films for effervescent muFDDSs should be strong and tough. In addition, they are expected to exhibit sufficient strain and resistance to rupture under high forces. Eudragit RL was the only single polymer that fulfilled all those requirements. However, a disadvantage of using Eudragit RL as the coating film to achieving pellet floating was the too-rapid release of the drug from this floating pellet system which should have a sustained-release pattern. Recently, it was reported that utilization of a combination of polymers with different physicochemical characteristics was able to overcome such limitations [21]. The optimized system of a mixture of Kollicoat SR with 5% each of triethyl citrate and polyethylene glycol (PEG) 600 at a 20% coating level began to completely float within 15 min and maintained its buoyancy over a period of 12 h with a sustained-release effect. However, none of those studies revealed the importance of mechanical properties of plasticized coating membrane on floating characteristics.

**Figure 1:**
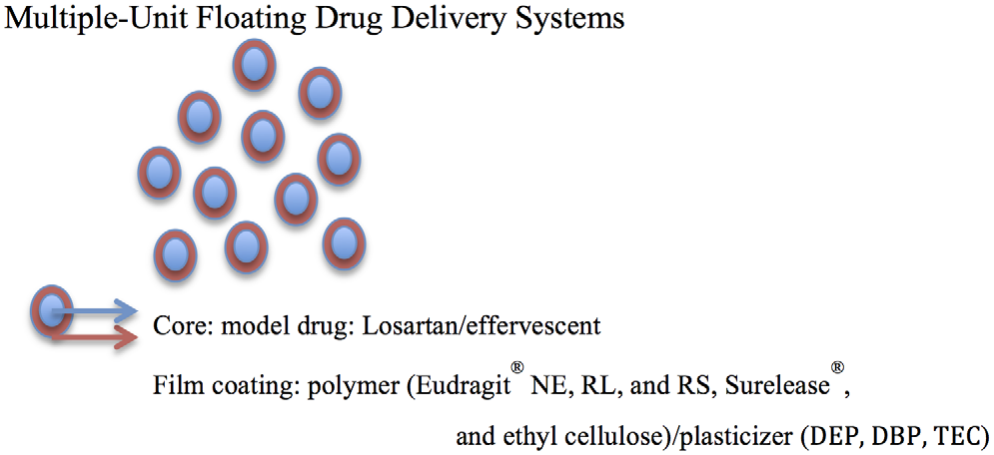
Schematic illustration of the structure of the effervescent multiple-unit floating drug delivery systems (muFDDSs).

In this study, plasticized polymeric membranes for preparing effervescent muFDDSs with controlled-release characteristics were characterized based on mechanical properties (stress, strain, modulus, and toughness). Water-insoluble polymeric films (Eudragit NE, RL, and RS, Surelease, and ethyl cellulose (EC)) combined with various plasticizers at different percentages were evaluated in both dry and wet states. Spherical core pellets containing the model drug losartan [24] and an effervescent agent (sodium bicarbonate) were prepared by an extrusion-spheronization process followed by coating with a plasticized polymeric film. Schematic presentation of the structure of the effervescent muFDDSs was shown in **Fig. 1**. The floating ability (floating lag time and duration) and drug-release profiles of the resulting muFDDSs were characterized.

## Materials and Methods

### Materials

Losartan potassium (IPCA, Bangalore, India) was chosen as the model drug. Sodium bicarbonate (NaHCO_3_, Merck, Darmstadt, Germany) was used as an effervescent agent to generate CO_2_ gas, and microcrystalline cellulose (MCCPH102, Wei Ming Pharmaceutical, Taipei, Taiwan) was a pelletization aid. All aqueous colloidal polymethacrylate dispersions (Eudragit NE, RS 30D, and RL 30D) were obtained from Evonik Industries AG (Essen, Germany). EC powder (10 cP) was supplied by Aqualon (Wilmington, DE, USA). Surelease (25% ethycellulose aqueous dispersion, E-7-19040) was purchased from Colorcon (Dartford Kent, UK). Diethyl phthalate (DEP, water solubility 0.928 g/L), dibutyl phthalate (DBP, water solubility 0.011 g/L) and triethyl citrate (TEC, water solubility 65 g/L) were provided by Merck. Hydroxypropyl methylcellulose 60SH-50 (HPMC 60SH-50, with a methoxy content of 28%?30%, a hydroxypropyl content of 7%?12%, and a viscosity of 2% solution in water of 50 cps), HPMC 60SH-4000 (HPMC 60SH-50, with a methoxy content of 28%?30%, a hydroxypropyl content of 7%?12%, and a viscosity of 2% solution in water of 4000 cps), and low-substituted hydroxypropylcellulose (LH-22) were obtained from Shin-Etsu (Tokyo, Japan). HPMC 90SH-K100M (with a methoxy content of 19%?24%, a hydroxypropyl content of 7%?12%, and a viscosity of 2% solution in water of 80,000?120,000 cps) and HPMC60SH-E10M (with a methoxy content of 28%?30%, a hydroxypropyl content of 7%?12%, and a viscosity of 2% solution in water of 7500?14,000 cps) were supplied by Colorcon. Microtalc (IT extra) was provided by Mondo Minerals (Amsterdam, the Netherlands). Tween 80 (polysorbate 80, Riedel-de Haën, Germany) was used as a dispersing agent. All other reagents were of analytical grade.

### Preparation and Characterization of Polymeric Films

Water-soluble plasticizers (TEC and DEP) and a water-insoluble plasticizer (DBP) were first thoroughly mixed in an aqueous solution and methanol, respectively. Then water-insoluble polymers (Eudragit RS and RL, and Surelease) were added as an aqueous dispersion at a final polymer level of 10% (w/w) and blended for 24 h for plasticization. In order to increase the water absorption, the powder form of EC dissolved in an 95% w/w of ethanol containing 20% of either of three plasticizers was further added with different grades of HPMC (60SH-50, 60SH-4000, E10M, and K100M) at 30% w/w with respect to the corresponding EC amount with or without extra water to completely dissolve the HPMC. Dried polymeric films were prepared by pouring the resultant mixture onto the parafilm-sealed bottom of a polyacrylic column, drying at 40°C for 8 h, and further curing at 50°C for 12 h. Wet films were prepared by soaking the dried polymeric films in 500 mL of simulated gastric fluid (SGF, 0.1 N HCl solution) at 37°C for 24 h. Polymeric films cut in a circle with a diameter of 30 mm were placed on a glass filter of a modified Enslin apparatus [25] at a temperature of 37°C. The water uptake profile was monitored by following the volume change reading from a graduated pipette with time (*n* = 4). The mechanical strengths of all polymeric films (10×3 mm with a thickness of 0.25?0.30 mm) were measured using a Dynamic Mechanical Analyzer (DMA7e, Perkin-Elmer, Waltham, MA, USA) by monitoring the time-modulus curve conducted at ambient temperature. The initial applied force was 5 mN, with an extension rate of 100 mN/min. The stress-strain curves for polymeric films were obtained, and the strain (%) and stress (MPa) at the rupture point were both recorded. The slope at the origin of the stress-strain curve gives Young’s modulus (MPa/%) and the total area under the stress-strain curve up to rupture is termed the modulus of toughness (MJ/m^3^) (*n* = ≥4) [26].

### Preparation and Floating Ability Test of Pellets

The pellet cores according to Table 1 were produced by extrusion (radial-basket type) through a screen size of 1 mm and spheronization at 700 rpm for 5 min (Shang Yuh Machine, New Taipei City, Taiwan), and the so-obtained pellets were dried at 40°C for 12 h. Since L-HPC was reported to be more advantageous in terms of water absorbability and swelling tendency than MCC, it was included in B formulations to examine its influence [27]. The coating solution was prepared by the same method as for polymeric films with a slight modification of adding 10% talc (w/w based on polymer solids) as an anti-adhesive to prevent coalescence during pellet coating. Pellets were coated using a rotor-type fluidized-bed system (GPCG-1, Glatt, Binzen, Germany) at respective optimal conditions (an inlet temperature of 44∼48°C, an outlet temperature of 34∼37°C, spray pressure of 15 psi, rotor speed of 180 rpm, and a flow rate of 4.5∼5.0 g/min) to a designated weight gain (% w/w). All coating efficiencies were found to exceed 90%. After coating, pellets were further cured at 50°C for 12 h, and pellets in the size range of 0.71?1.25 mm were collected for further experiments. A detailed formulation design of pellets and coating films and their respective code names are given in Table 2.

**Table 1:** Pharmaceutical composition of the core pellets.

	A	B	A20	C20
Losartan (mg)	50	50	50	50
MCC (mg)	200	100	150	75
L-HPC (mg)		100		75
NaHCO_3_ (mg)			50	50
Total (mg)	250	250	250	250

**Table 2:** Pharmaceutical composition of floating pellets with a film coating.

Formulation	Coating layer^a^			Core pellets
	Polymer	Plasticizer^b^	Coating level (%)	
A-RS TEC 15 10%	Eudragit RS	TEC 15%	10	A
A-RS TEC 15 20%		TEC 15%	20	A
B-RS TEC 15 3%		TEC 15%	3	B
B-RS TEC 15 6%		TEC 15%	6	B
B-RS TEC 15 9%		TEC 15%	9	B
B-RS TEC 15 12%		TEC 15%	12	B
A20-RS TEC 15 10%		TEC 15%	10	A20
A20-RS TEC 15 15%		TEC 15%	15	A20
A20-RS TEC 15 20%		TEC 15%	20	A20
A20-RL TEC 15 10%	Eudragit RL	TEC 15%	10	A20
A20-RL TEC 15 20%			20	A20
A20-RL TEC 15 30%			30	A20
A20-RL TEC 15 40%			40	A20
A20-RL DEP15 20%		DEP 15%	20	A20
A20-RL DEP15 30%			30	A20
A20-RL DEP15 40%			40	A20
A20-RL DBP15 20%		DBP 15%	20	A20
A20-RL DBP15 30%			30	A20
A20-RL DBP15 40%			40	A20
A20-1S1LDEP15 10%	Eudragit RL: RS = 1∶1	DEP 15%	10	A20
A20-1S1LDEP15 15%			15	A20
A20-1S1LDEP15 20%			20	A20

a Diluted with water (the final suspension had a concentration of 10% of solid polymer and plasticizer) and the addition of 10% talc (w/w based on polymer solids).

b w/w based on polymer solids.

For the floating study, 100 pellets were placed in the medium (SGF, 0.1 N HCl), and the time for all pellets to float (TPF) and floating duration (the duration when a certain percentage had pellets floated (%)) were determined by visual counting. The TPF was defined as the time that 100 pellets completely floated to the top surface of 900 ml of SGF at 37°C and a stirring rate of 50 rpm. The percentage of floating pellets was defined as the floating pellets (%) based on the following equation (1):
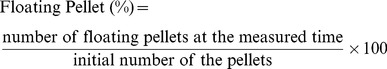
(1)


### Drug-Release Studies

Drug dissolution from the coated pellets was conducted in 900 mL of SGF at 37±0.5°C and 50 rpm based on the apparatus II method (USP XXIX) (VK7020, Agilent Technologies Inc, Santa Clara, CA, USA). The medium (5 mL) was sampled at predetermined times and replaced with fresh medium of the same volume. The drug concentration was measured with an ultraviolet/visible spectrophotometer (V-550, Jasco, Tokyo, Japan) at a wavelength of 254 nm that had been validated to have acceptable precision and accuracy. Each *in* *vitro* release study was performed in at least triplicate.

### Theoretical Consideration of the Minimal Strain of Polymeric Films to Make Pellets Floats

At equilibrium of floating pellets, the magnitude and direction of the force, *F*_net_, corresponded to the vectorial sum of the buoyancy (*F*_buoy_) and gravitational (*F*_grav_) forces acting on the pellet [28]. By definition, the more positive *F*_net_ is (forces directed upward), the faster and longer a pellet floats. As described by the following equation, where *F*_net_ is the net vertical force, *g* the acceleration of gravity, *d*_F_ the fluid density, *d*_P_ the pellet density, *M* the pellet mass, *V*_P_ and *V*_SP_ the pellet volume before and after swelling, respectively, and ε_v_ and ε_d_ the strain of pellet volume and pellet diameter, respectively:

*F*_net_ = *F*_buoy_−*F*_grav_ = *d*_F_·*g*·*V*_SP_–*d*_P_·*g*·*V*_P_ = *d*_F_·*g*·ε_v_·*V*_P_–(*M*/*V*_P_)·*g*·*V*_P_

 = (*d*_F_·ε_v_·*V*_P_–*M*)·*g*

*= *(*d*_F_·ε_v_·(*M*/*d*_P_)–*M*)·*g*

*= *(*d*_F_·ε_v_/*d*_P_–1)·*M*·*g*.

To be able to float, *F*_net_ must be greater than zero. Assuming *d*_F_ = 1.0 for the water medium, the minimal value for the strain of pellet volume (ε_v_) and the strain of pellet diameter (ε_d_) can be predicted as related to the pellet density (*d*_P_:

*d*_F_·ε_v_/*d*_P_ ≥ 1.0

ε_v_ ≥ *d*_P_ and ε_d_ ≥ (*d*_P_)^1/3^.

**Figure 2:**
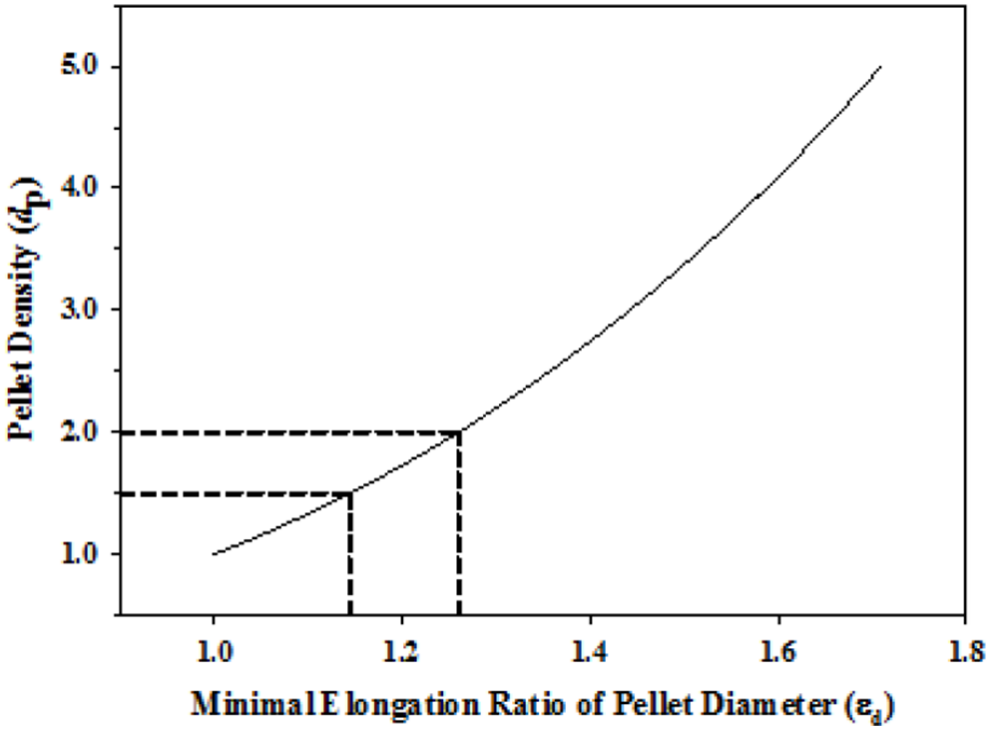
Theoretical plot of the pellet density versus the minimal elongation ratio of the pellet diameter based on (ε_d_ =  (*d*_P_)^1/3^).

Based on Eq. 3, a theoretical plot of *d*_p_ in the range of 1.0?5.0 versus the corresponding minimal ε_d_ (ε_d_ =  (*d*_P_)^1/3^) is illustrated in **Fig. 2**. It demonstrates that for a pellet with the density of 1.5 or 2.0, its minimal elongation of polymeric thin film to make pellet floating is about 14% or 26%, respectively.

### Statistical Analysis

The analysis was done with a linear regression (PASW Statistics 18.0, Chicago, IL, USA) to assess the relationship between independent variables (water permeability or strain in the wet state) with all dependent variables (TPF, floating duration, and drug-release rate) of different formulations by multi-variant analysis with a stepwise regression. A *p* value of <0.05 was considered statistically significant.

## Results and Discussion

### Preparation and Characterization of Polymeric Films

**Figure 3:**
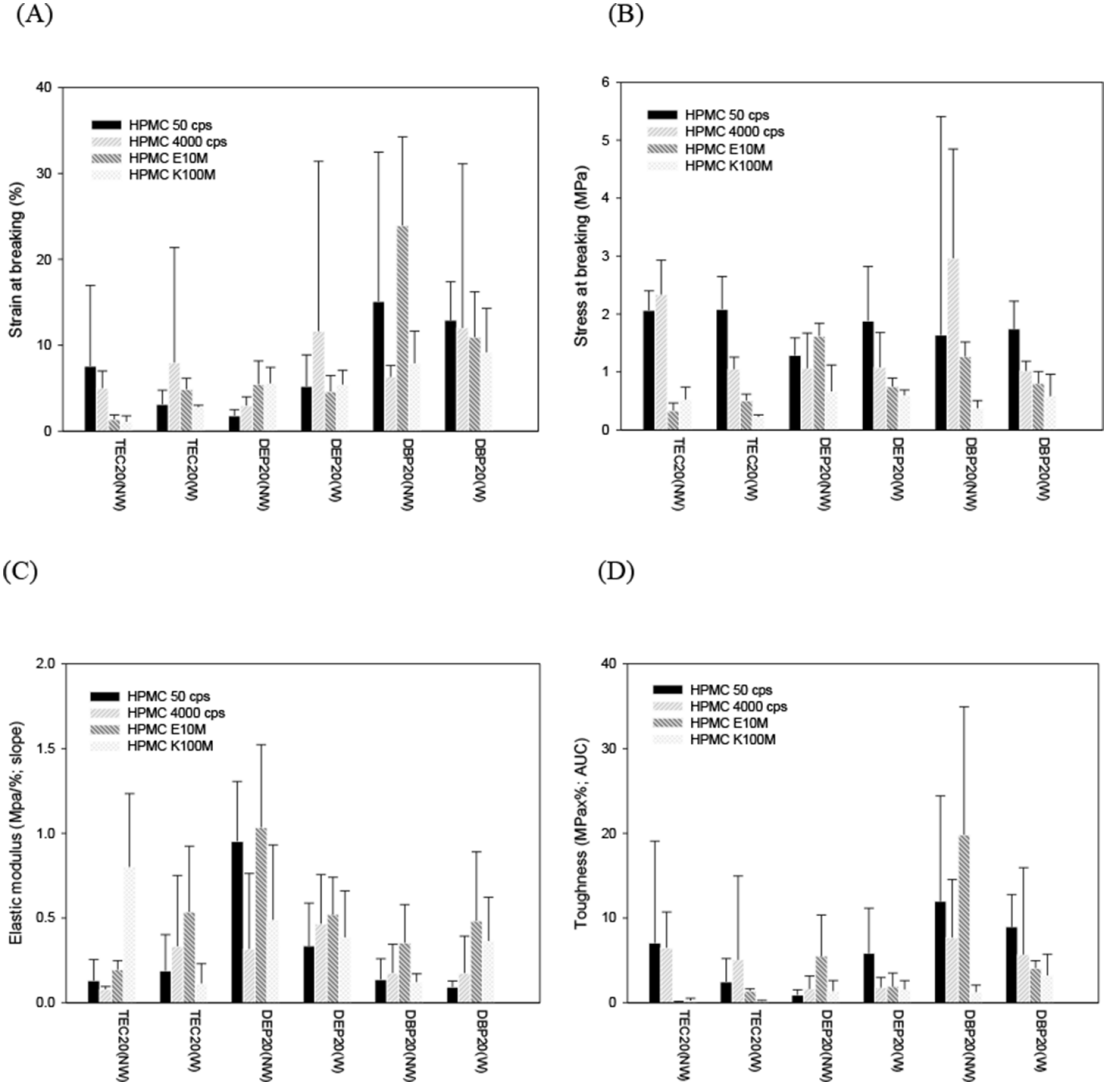
Mechanical properties of wet-state films composed of ethyl cellulose and different grades of HPMC. (A) Strain at breaking (%), (B) stress at breaking (MPa), (C) elastic modulus (MPa/%; slope), (D) toughness (MPa×%; AUC) (*n* = 4).

An ideal coating film on pellets for floating DDSs requires that (1) the water permeability of the coating film should be fast enough to generate enough CO_2_ gas for a buoyancy effect to make the pellet float, with sooner being better; (2) the flexibility of the coating film should be sufficient to withstand disruptive forces exerted by the CO_2_ gas generated inside the pellet core; and (3) the permeability of the CO_2_ gas generated across the coated film should be limited to maintaining a longer duration of floating. Since plasticizers play an important role in determining not only the water permeability and flexibility of polymeric film but also the permeability of CO_2_. The influences of the three most commonly used plasticizers (TEC, DEP, and DBP) added at various levels on the mechanical properties of water insoluble polymers (Eudragit RS, RL, NE, Surelease, and EC) were examined. The stress-strain curve for polymeric films was obtained by applying a tensile force at a uniform rate and constant temperature. The mechanical properties measured included Young’s modulus (slope at the origin, *E*), stress at break (*σ*), elongation (strain) at break (*ε*), and the modulus of toughness of the polymeric films (the total area up to break, *T*) for polymeric coating films are listed in Tables 3–5 for Eudragit RS, RL, and Surelease (EC aqueous dispersion, EC_sure_), respectively, and is illustrated in Fig. 3 for EC (dissolved in 95% w/w of ethanol, EC_sol_).

**Table 3:** Mechanical properties of dry and wet Eudragit RS films to which different plasticizers were added (mean±SD; *n* = 4).

	Strain at breaking (%)	Stress at breaking (MPa)	Elastic modulus (MPa/%; slope)	Toughness (MPa×%; AUC^a^)
**Dry state**				
TEC15	446.47±229.41	3.0039±0.4342	0.1239±0.0169	989.68±594.70
TEC30	786.30±181.64	0.4139±0.1560	0.0030±0.0006	211.88±157.09
DEP15	690.75±113.42	2.2363±0.3941	0.0743±0.0341	1129.6±379.37
DEP30	299.64±27.43	0.5430±0.0484	0.0076±0.0025	73.306±15.436
DBP15	434.27±279.76	4.1246±0.6028	0.1512±0.0543	1247.1±915.99
DBP30	558.33±289.40	0.6571±0.0975	0.0053±0.0029	222.10±152.37
**Wet state**				
TEC15	314.86±81.359	1.5674±0.0759	0.0520±0.0190	246.24±100.07
TEC30	157.77±56.59	0.8202±0.1402	0.0265±0.0102	63.989±24.066
DEP15	203.31±21.52	0.9995±0.0406	0.0573±0.0625	114.74±16.126
DEP30	363.94±133.48	0.5906±0.0872	0.0142±0.0075	109.59±57.422
DBP15	683.17±415.88	1.0441±0.2547	0.0198±0.0099	429.30±288.65
DBP30	485.74±205.13	0.3493±0.1106	0.0061±0.0024	106.97±72.246

a AUC: area under curve.

**Table 4:** Mechanical properties of dry and wet Eudragit RL films to which different plasticizers were added (mean±SD; *n* = 4).

	Strain at breaking (%)	Stress at breaking (MPa)	Elastic modulus (MPa/%; slope)	Toughness (MPa×%; AUC)
**Dry state**				
TEC15	580.14±122.55	2.1692±1.1637	0.0412±0.0284	862.52±570.94
TEC30	797.47±124.37	0.3895±0.0550	0.0041±0.0018	183.31±38.917
DEP15	601.88±66.87	3.1674±1.4374	0.0659±0.0164	470.12±301.73
DEP30	66.46±21.46	0.4918±0.1306	0.0092±0.0018	63.841±78.045
DBP15	109.81±36.13	3.6164±0.3243	0.0766±0.0253	259.74±136.77
DBP30	282.84±38.11	0.5294±0.4627	0.0068±0.0058	76.560±69.535
**Wet state**				
TEC15	226.50±167.23	2.5874±0.9953	0.1612±0.1111	480.60±545.52
TEC30	221.79±146.83	1.5295±0.2939	0.0867±0.0408	257.40±267.62
DEP15	411.46±275.43	1.5275±0.5158	0.0947±0.1314	525.01±505.54
DEP30	115.02±79.574	0.4414±0.4031	0.0147±0.0161	44.717±70.662
DBP15	431.97±211.87	0.3773±0.1334	0.0011±0.0012	93.873±78.816
DBP30	642.82±324.24	0.0845±0.0181	0.00015± 0.0003	15.443±7.7931

**Table 5:** Mechanical properties of dry and wet Surelease films to which different plasticizers were added (mean±SD; *n* = 4).

	Strain at breaking (%)	Stress at breaking (MPa)	Elastic modulus (MPa/%; slope)	Toughness (MPa×%; AUC)
**Dry state**				
TEC15	6.7361±2.8417	0.8079±0.2859	0.1788±0.0326	2.7533±2.3976
TEC30	16.523±8.0149	0.6695±0.2050	0.0678±0.0287	6.6291±4.0169
TEC40	29.101±13.149	0.4456±0.0765	0.0192±0.0083	6.4249±4.9425
DEP15	24.903±2.2796	0.8544±0.0708	0.0755±0.0216	10.784±3.3239
DEP30	47.954±28.124	0.1863±0.0467	0.0084±0.0023	4.6311±2.9695
DEP40	48.567±30.050	0.1056±0.0678	0.0033±0.0036	1.8308±1.5193
DBP15	48.190±13.677	0.9065±0.0523	0.0583±0.0280	24.029±12.256
DBP30	59.332±26.369	0.1763±0.0432	0.0051±0.0041	5.9398±4.6835
DBP40	105.18±81.586	0.1297±0.0336	0.0027±0.0007	6.5892±5.9964
**Wet state**				
TEC15	13.645±5.7402	1.1292±0.2477	0.1294±0.0619	6.5684±5.4072
TEC30	27.250±11.057	1.1020±0.4379	0.0706±0.0387	15.468±16.032
DEP15	23.583±6.0892	1.5542±0.3462	0.3647±0.3092	20.372±10.613
DEP30	79.160±19.205	1.9423±0.7567	0.0984±0.0760	91.969±60.517
DBP15	52.389±14.903	0.3327±0.0456	0.0114±0.0067	7.6282±4.5475
DBP30	118.53±56.976	0.0999±0.0473	0.0014±0.0013	4.7794±5.3844

As shown in Tables 3 and 4, respectively, both Eudragit RS and RL films measured in the dry state demonstrated that *ε* increased with an increasing addition level of all three plasticizers (except DEP as a plasticizer), whereas *E*, *σ*, and *T* decreased with an increasing addition level. Among the three plasticizers used, TEC expressed the highest plasticizing effect to cause a greater extent of elongation. However, when mechanical properties of both Eudragit RL and RS films were measured in the wet state, the plasticizing effect of water obviously decreased their mechanical strengths. Further, adding TEC as plasticizer caused a greater decreasing extent of *ε* in the wet state, and also resulted in a less elongation tendency compared to the dry state. This phenomenon is comparable to that reported by Bodmeier and Paeratakul [27]. It was due to differences in the water solubilities among the three plasticizers. Since TEC has a water solubility of 65 g/L, a greater amount of TEC could be released in comparison to the two other plasticizers when soaking polymeric films in the GIF medium leading to a disappearance of the plasticizing effect of TEC. Further, the decreasing extent of a plasticizing effect of TEC was expected to be more obvious when the level added increased from 15% to 30%. Regarding DEP, less influence on the plasticizing effect was seen at an addition level of 15%, whereas a profound decrease in *ε* from 690.75% and 601.88% to 299.64% and 66.46% for Eudragit RS and RL, respectively, was observed at the 30% addition level. This seems to indicate that such a release amount of DEP was still able to have a greater influence on mechanical properties when the added amount of DEP was higher. Further, at both 15% and 30% addition levels of DBP in Eudragit RS and RL films, an insignificant change in mechanical properties was seen when measured in the wet state. Obviously, this can be explained by there being the least amount of DBP released from the polymeric film during soaking since DBP is only slightly soluble in water. It was concluded that the volume of plasticized RS- and RL-coated pellets might be expanded without rupture under the tensile stress (which should be equal to or greater than the tensile stress that could be exerted by a 5-mN force applied by DMA) exerted by the CO_2_ gas pressure which generated sufficient buoyancy to make the pellets float.

Compared to the Eudragit RS film, the RL film was more highly permeable to water because it was composed of a higher fraction of hydrophilic quaternary ammonium groups in the structure. This higher water permeability not only made RL film able to absorb more water to enhance the plasticizing effect, but also made water-soluble plasticizers leak at a greater amount and a faster rate from RL film than water-insoluble plasticizers. Hence, a greater extent of changes presented in mechanical properties between measurements in the dry and wet states for water-soluble plasticizers. In addition, a higher permeability of RL membranes to water should enable RL-coated pellets to generate CO_2_ gas faster, leading to a shortened TPF.

Surelease is a commercially available product containing an EC aqueous dispersion at a 25% w/w solids content and already optimally plasticized with medium-chain triglycerides (MCTs). The same as shown by Table 5, EC films prepared from Surelease (EC_Sure_) showed a similar tendency for the influence of the three plasticizers at two different levels on their mechanical properties as that of Eudragit RS and RL in both the dry and wet states. However, the strain (%) of EC_Sure_ films so prepared regardless of which plasticizer was used at two levels were observed to be obviously lower than those of corresponding formulations of both Eudragit RL and RS. The intermolecular hydrogen bonds and sugar-based three-dimensional hindrances might have resulted in the poor extendibility of EC. This was also confirmed by Sungthongjeen et al. [15], who determined that the EC_Sure_ membrane is a mechanically weak and brittle polymer and not flexible and hence can easily rupture once CO_2_ pressure is generated. This means that the volume expansion of EC_Sure_-coated pellets under stress exerted by the CO_2_ gas generated might not be able to reach a significant extent with a sufficient buoyant force to make the pellets float.

When the polymeric film of Eudragit NE without adding any plasticizer was measured in both the dry and wet states, it was found that polymeric film of Eugragit NE was already too ductile to break (over the instrument limit of static strain, 1064.82%) (data not shown). This is because Eudragit NE is a neutral ester polymer (ethyl acrylate and methyl methacrylate in a 2∶1 ratio) with no hydrogen bonds or other intermolecular forces, and the glass transition temperature (*T*_g_) value is approximately 5°C; therefore, it has high flexibility (static strain >600%) at room temperature. Our results were consistent with those reported by Sungthongjeen et al. [15], for which an Eudragit NE film was observed to have the highest elongation values in both the dry and wet states. This polymer dispersion has a low minimum film formation temperature and does not require plasticizers, resulting in flexible films [23]. In the wet state, the strain of Eudragit NE films decreased to a lesser extent than that in the dry state. This can be explained by the hydrophobic character of Eudragit NE [29]. It was concluded that the lower permeability of Eudragit NE for water due to its hydrophobic character might be a hindrance to generating CO_2_ gas faster for shortening the TPF although the volume expansion of NE-coated pellets might be large enough to generate sufficient buoyancy to promote pellet floating.

Table 6 illustrates the mechanical properties of EC films (EC_sol_) cast from the 95% w/w of ethanol supplemented with either one of three plasticizers (TEC, DEP, and DBP) at the 20% level and 30% (w/w, with respect to the EC amount) of various grades of HPMC as a water-permeation enhancer with or without a sufficient quantity of water (W/NW) to dissolve the added HPMC. Results demonstrated that except for the static strain of the EC_sol_ film (NW-DBP20) plasticized with DBP and incorporating HPMC E10M without adding water which was around 30%, those for the other EC_sol_ films remained <10%. This indicates that the addition of various grades of HPMC was unable to enhance the flexibility of EC_sol_ membranes to accommodate pellet expansion.

**Table 6:** Diameter and volume changes of pellets after immersion in gastric fluid.

Formulation	Pre-immersion (0 min)		After 1 h	
	Diameter(mm)	Volume(ml)	Diameter(mm)	Volume(ml,×10^4^)
A-RS TEC 15 10%	1.05±0.07	6.06×10^−4^	NF	
A-RS TEC 15 20%	1.09±0.10	6.78×10^−4^	NF	
B-RS TEC 15 3%	1.00±0.05	5.24×10^−4^	NF	
B-RS TEC 15 6%	1.03±0.04	5.72×10^−4^	NF	
B-RS TEC 15 9%	1.02±0.04	5.56×10^−4^	NF	
B-RS TEC 15 12%	1.05±0.06	6.06×10^−4^	NF	
A20-RS TEC 15 10%	1.07±0.06	6.41×10^−4^	NF	
A20-RS TEC 15 15%	1.09±0.06	6.78×10^−4^	NF	
A20-RS TEC 15 20%	1.12±0.09	6.34×10^−4^	NF	
A20-RL TEC15 20%	1.03±0.11	5.72×10^−4^	1.62±0.20	22.26
A20-RL TEC15 30%	1.01±0.08	5.39×10^−4^	1.34±0.09	12.60
A20-RL TEC15 40%	1.05±0.07	6.06×10^−4^	1.55±0.16	19.50
A20-RL DEP15 20%	1.01±0.09	5.39×10^−4^	2.22±0.19	57.29
A20-RL DEP15 30%	1.06±0.12	6.24×10^−4^	2.80±0.20	114.94
A20-RL DEP15 40%	1.10±0.05	6.97×10^−4^	2.69±0.28	101.92
A20-RL DBP1520%	1.11±0.04	7.16×10^−4^	2.39±0.23	71.48
A20-RL DBP15 30%	1.14±0.06	7.76×10^−4^	2.50±0.24	81.81
A20-RL DBP15 40%	1.13±0.05	7.55×10^−4^	2.67±0.31	99.67
A20-1S1L DEP15 10%	1.06±0.12	6.24×10^−4^	1.94±0.10	38.23
A20-1S1L DEP15 15%	1.06±0.05	6.24×10^−4^	2.07±0.17	46.44
A20-1S1L DEP15 20%	1.08±0.05	6.60×10^−4^	1.97±0.15	40.03

### Water-Uptake Rate and Amount

**Figure 4:**
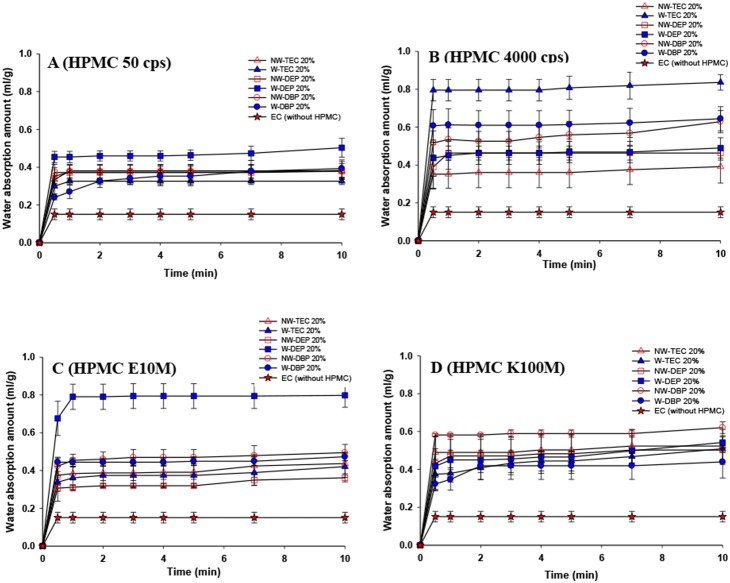
Water uptake plots of ethyl cellulose film samples plasticized with various plasticizers at 20% w/w and after adding 30% w/w of (A) HPMC 50 cps, (B) HPMC 4000 cps, (C) HPMC E10M, (D) HPMC K100M (*n* = 4).

Permeation of a liquid through the polymeric film into the core and the subsequent generation of CO_2_ may play major roles in the floating and drug-release characteristics. The gastric emptying time ranges from 15 min to 3 h, so FDDSs should float within 15 min. The water uptake rate was evaluated for EC_sol_ films with various grades of HPMC added at 30% w/w with respect to the EC weight, and results are illustrated in **Fig. 4**. It demonstrates that the addition of any grade of HPMC led to faster uptake of greater amounts of water. An insignificant effect of adding water to make HPMC completely dissolve was seen on the water-uptake rate and amount taken up.

### Preparation and Floating Characterization of Coated Pellets

**Figure 5:**
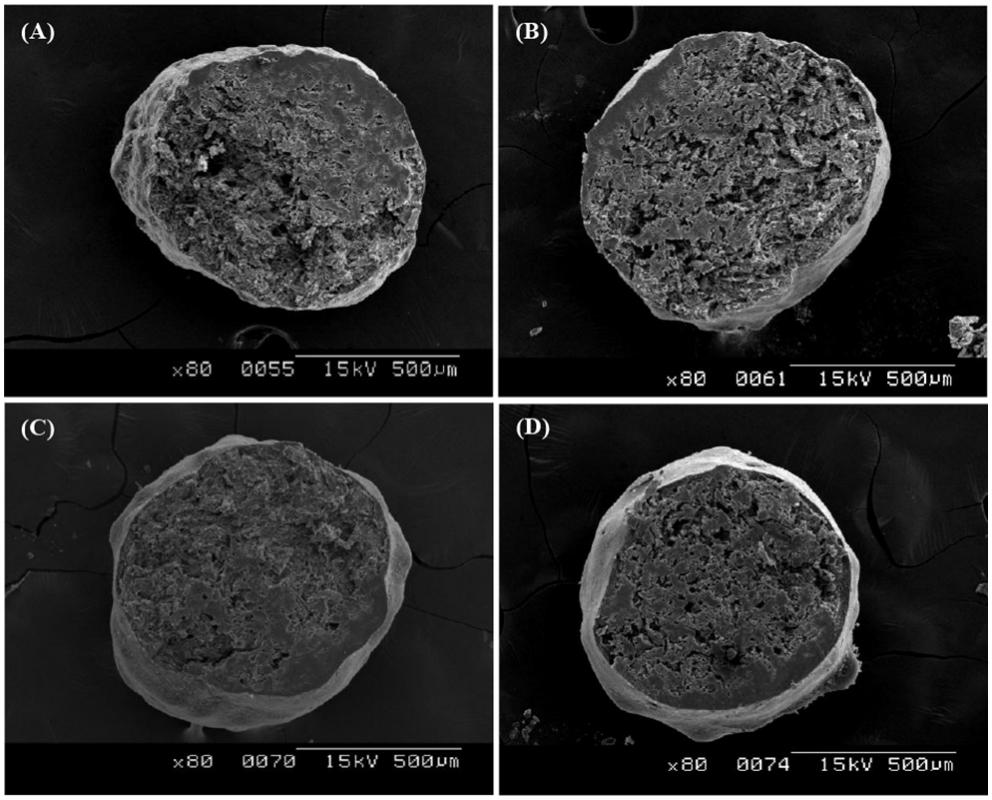
SEM photos of pellets (B-RS TEC15) at various coating levels (w/w) of (A) 3%, (B) 6%, (C) 9%, and (D) 12%.

The muFDDS systems consisted of a drug (lorsartan)-containing core pellet with an effervescent (NaHCO_3_) and pelletization aid (MCC), and a gas-entrapping polymeric membrane. Developing a successful effervescent muFDDS requires rapid formation of a low-density system within minutes after contact with gastric fluid and maintenance of the buoyancy in the stomach with controlled release. The integrity of coated films on the pellet surface with uneven texture would be a prerequisite for achieving these goals. SEM photographs shown in **Fig. 5** demonstrate that the integrity of coated films improved with an increasing coated level. It clearly illustrates that at least a 9% coated level is required to fully cover with an uneven surface of pellets produced by the extrusion-spheronization process (B-RS TEC15). Therefore, a coating level of >10% was selected for all coated polymeric films in the following experiment.

### Floating Ability

**Figure 6:**
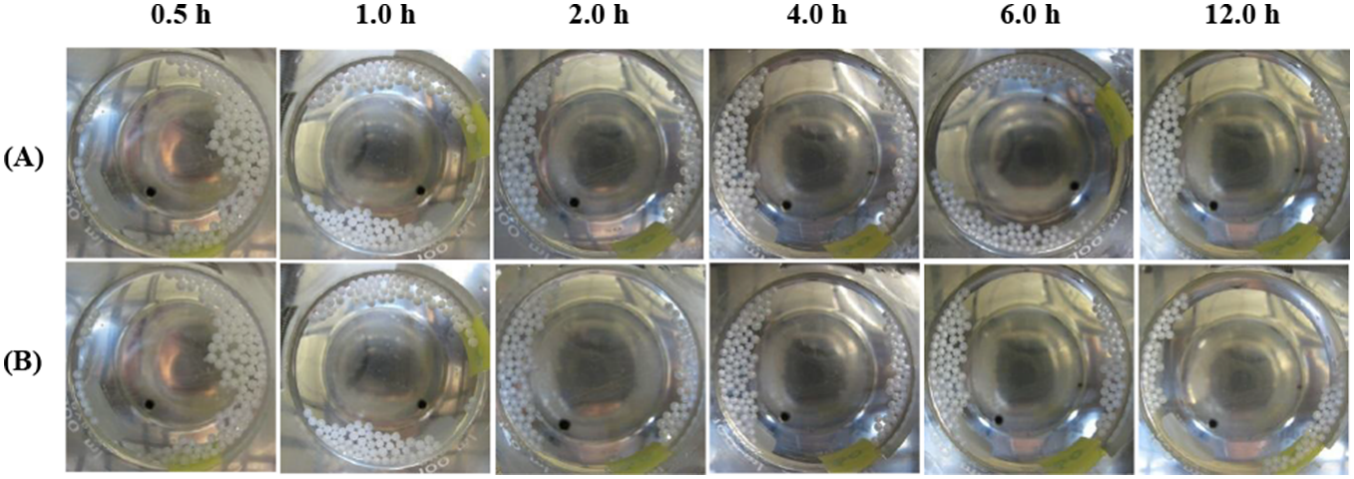
Photographs of pellets (A20-RL DEP15 20%) (A) and (A20–1S1L DEP15 20%) (B) after immersion in pH 1.2 buffer for specific times.

As shown in **Table 6**, when using Eudragit RS 30D as the coating polymer with 15% TEC as the plasticizer, core A pellets (MCC as pelletization aid) coated at the 10% and 20% levels, core B pellets (MCC+L-HPC as pelletization aid) coated at 3%∼12%, and core A20 pellets (NaHCO_3_ for gas generation) coated at 10%∼20% were found to be unable to float during the 24-h observation period. However, when using Eudragit RL 30D as the coating polymer on A20 core pellets, TEC and also DEP and DBP could make the pellets float within 20 min in 0.1 N HCl medium even at a coating level of as high as 40%. This result conforms to that reported by Sungthongjeen et al. [15]. It was attributed to the difference in the permeability of the acidic medium and the extent of elongation as a result of expansion of the CO_2_ gas generated when NaHCO_3_ was neutralized by the inward-permeating acidic medium. As predicted by **Fig. 2**, coated pellets must expand to some extent in order to increase the volume, which in turn decreases the density of the pellets to make them floatable as a result of a more-positive upward *F* (the vectorial sum of the *F*_buoy_ and *F*_grav_ forces). Expansion of the pellets was observed for those floating on the surface of 0.1 N HCl medium as shown by **Fig. 6A and 6B** respectively for pellets (A20-RL DEP15 20%) coated with Eudragit RL and those (A20-1S1L DEP15 20%) coated with Eudragit RL:RS at 1∶1. Those photographs clearly illustrate that there is a white core suspended inside the pellet and a translucent space between the coated membrane and center core, which was apparently created by the expansion of the CO_2_ gas generated. This phenomena was not observed when Eudragit RS was used as the coating polymer (data not shown), and we found that no pellets coated with Eudragit RS were able to float. It was concluded that Eudragit RL coating films, but not Eudragit RS, were able to allow a faster permeation of the acidic medium into the pellets. This resulted in elongation of the Eudragit coating films under outward expansion of the CO_2_ gas generated from neutralization of the inward-permeating acidic medium with NaHCO_3_. Consequently, sufficient CO_2_ then make the pellets less dense and thus to float.

**Figure 7:**
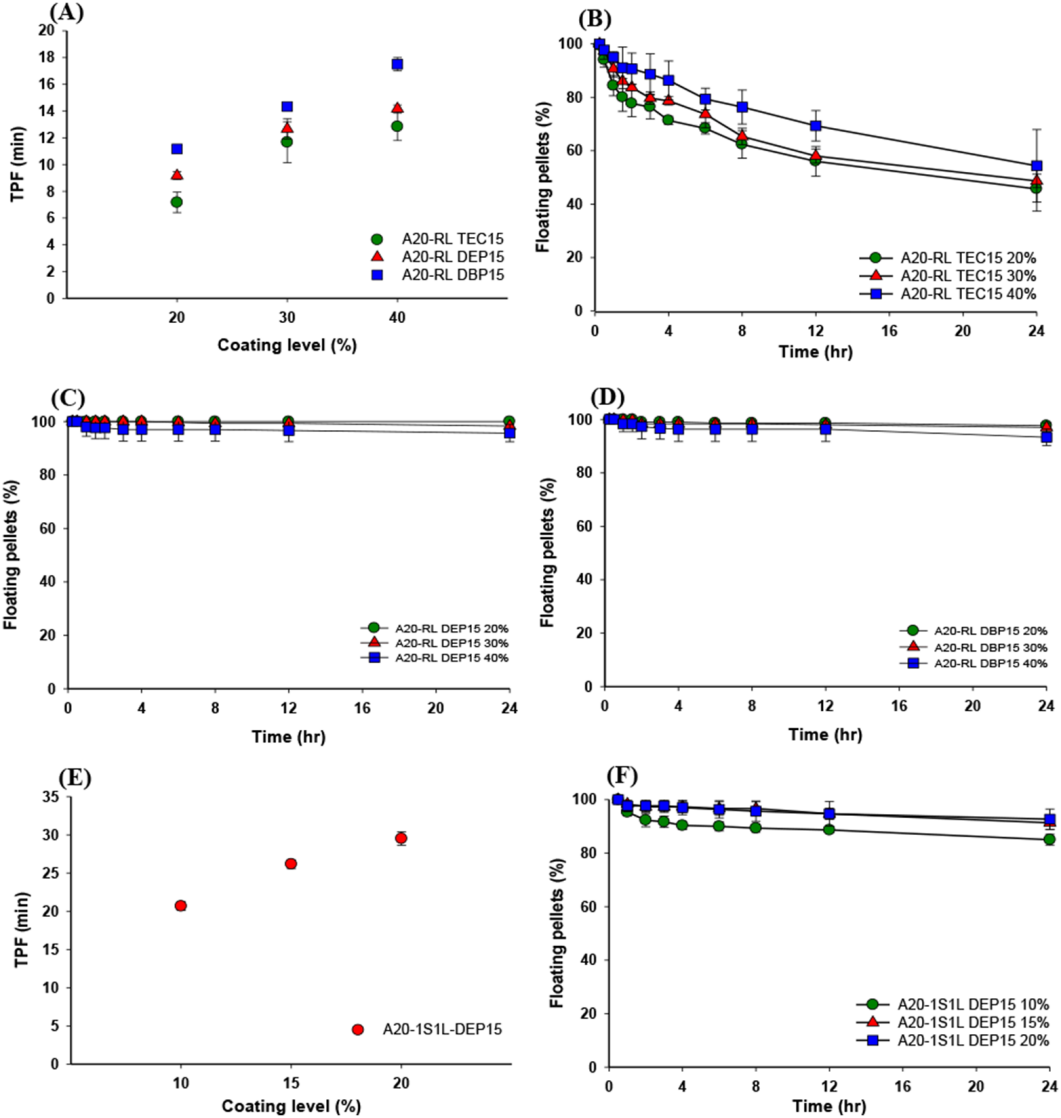
Effect of the coating level of Eudragit RL 30D (A) or Eudragit RL 30D RS 30D at 1∶1 (E) and different plasticizers on the time to float of pellets in pH 1.2 buffer at 37°C. Floating patterns of the pellets coated with Eudragit RL 30D (B, C, and D) or Eudragit RL 30D: Eudragit RS 30D at 1∶1 (F) after immersion in pH 1.2 buffer at 37°C.

Results of the TPF measurements and the percentage of floating pellets for both Eudragit RL and Eudragit RS:RL at 1∶1 coating films with different plasticizers at various levels on A20 core pellets are shown in **Fig. 7**. As shown in **Fig. 7A and 7E**, TPFs for both coating films increased with an increasing coating level for all plasticizers examined. Also **Fig. 7A** further indicates that the lower solubility of the plasticizer in water was (TEC>DEP>DBP), the longer the TPF results were (DBP>DEP>TEC). Theoretically, the water permeation rate per unit area (d*Q*/(A*d*t*) = D*Δ*C*/*h*) is proportionally inverse to the thickness of the coating film (*h*) and is proportional to the diffusion coefficient (*D*), which could be influenced by the hydrophilicity of the coated membrane. Since increasing the thickness of coating films with an increasing coating level theoretically leads to a decrease in the water-permeation rate, this results in the slower generation of CO_2_ gas to expand the pellet such that it has a density low enough to float and in turn a longer TPF. The addition of hydrophobic plasticizers within the coated films creating a more-hydrophobic environment for water permeation probably decreases the diffusion coefficient of water in those plasticized coated films. As a result, the lower permeation rate of the acidic medium through the coated films due to a decreasing diffusion coefficient with increasing hydrophobicity of the added plasticizer more likely causes slower generation of CO_2_ gas to expand the pellet until it has a density low enough to float and in turn a longer TPF.

Furthermore, **Fig. 7B–7D** demonstrate the time course of percent pellets floating for Eudragit RL coating films plasticized with 15% TEC, DEP, and DBP, respectively, at three coating levels (20%, 30%, and 40%), and **Fig. 7F** illustrates Eudragit RS:RL at 1∶1 coating films plasticized with 15% DEP at three coating levels (10%, 15%, and 20%). When comparing among the percent pellet floating time plots for Eudragit RL coating films plasticized with three different plasticizers (**Fig. 7B**, TEC; **Fig. 7C**, DEP, and **Fig. 7D**, DBP), almost all pellets coated with Eudragit RL plasticized with DEP and DBP could maintain floating for as long as 24 h, whereas the percent pellet floating for pellets coated with Eudragit RL films plasticized with TEC decreased with increasing time. As observed by **Fig. 7B**, it was found that the percent pellets floating for pellets coated with Eudragit RL plasticized with 15% TEC at three different coating levels all decreased with increasing time. The extent of percent pellets floating at the same time point decreased with a decreasing coating level (20%>30%>40%), and the corresponding extents of percent pellets floating at the end of 24 h were 47%, 49%, and 54%, respectively for coating levels of 20%, 30%, and 40%. However, **Fig. 7C and 7D** show that using Eudragit RL plasticized with DEP and DBP as the coating films could maintain pellets floating for as long as 24 h, and even when increasing the coating level from 20% to 30%, and even further to 40%, the pellets still maintained their floatability. All of these phenomena might be attributed to differences in water solubility. TEC with the highest solubility in water could make the permeation rate of acidic medium faster in the initial period to shorten the TPF as described above. But the higher water solubility could also cause a greater amount of TEC to be released with increasing time resulting in the decay of its plasticized effect on the coated film and in turn a gradual loss of its floatability over time. However, both DEP and DBP have lower water solubilities and are released at such a lower level that they could not deteriorate the plasticized effect on the coated film and in turn effectively maintained the floatability of pellets over time. Overall, it was concluded that a plasticizer like DEP with its water solubility is optimal for use in combination with Eudragit RL as the coated film on pellets to produce an appropriate TPF of as short as 15 min and a floating period as long as 24 h.

**Table 6** further compares the expansion extents of the pellet diameter and volume after immersion in 0.1 N HCl medium for 1 h (the time to reach equilibrium of expansion) with that of pellets before immersion for various coated films with different plasticizers added at the same 15% level. It reveals that all three core pellets (A, B, and A20 cores) coated with Eudragit RS with 15% of TEC as the plasticizer were unable to expand after immersion leading to no pellets floating. On the contrary, Eudragit RL with the same 15% TEC as the plasticizer coated on A20 core pellets was able to allow acidic medium to permeate into the pellet to generate CO_2_ gas for expansion and then floating. When two other plasticizers, DEP and DBP, were added at the same 15% level, an expansion of the pellet volume was observed but to different extents. In comparison to the extent of volume expansion after 1 h of immersion, 4-, 11-, and 10-fold expansions of the pellet volume were observed when using TEC, DEP, and DBP as the plasticizer, respectively, at the same 20% coating level of Eudragit RL. This clearly shows that the expansion of coated Eudragit RL films was the least when using TEC as the plasticizer, and greater extents of increases in volume expansion occurred when using DEP and DBP as plasticizers. These data further confirm that the plasticized effects of both DEP and DBP in a wet state are greater than that of TEC leading to a greater extent of elongation as discussed above. These data also support longer floating periods when using DEP and DBP as plasticizers than when using TEC.

In order to search for alternative coated films suitable for muFDDSs, a combination of Eudragit RS:RL at 1∶1 was examined using DEP as the plasticizer in consideration of its effect on the TPF and floating period. As illustrated in **Fig. 7E**, all pellets coated with Eudragit RS:RL at 1∶1 with 15% DEP as the plasticizer at the 10% coating level were observed to float within 20 min (TPF). However, when increasing the coating level to 15% and 20%, TPFs were prolonged to 25 and 30 min, respectively. A 6?7-fold increase in pellet volume after 1 h of immersion in 0.1 N HCl was observed for those pellets with the same coating film at a coating level of 10%?20% as shown in **Table 6**. However, maintaining a longer floating period was observed for those pellets with the same coating film at a coating level of 10%?20% as shown in **Fig. 7F**. Prolongation of the TPF and a lower extent of volume expansion were expected for pellets coated with Eudragit RS:RL at 1∶1 since a lower permeability of water and a lower elongation for Eudragit RS than that for Eudragit RL occurred with the same plasticizer and added amount. Nevertheless pellets coated with Eudragit RS:RL at 1∶1 (15% DEP) at the 10% level still achieved 86% pellets floating for 24 h. Therefore, this film composition could be an alternative to Eudragit RL as a coating film for muFDDSs.

### Drug-Release Studies

**Figure 8:**
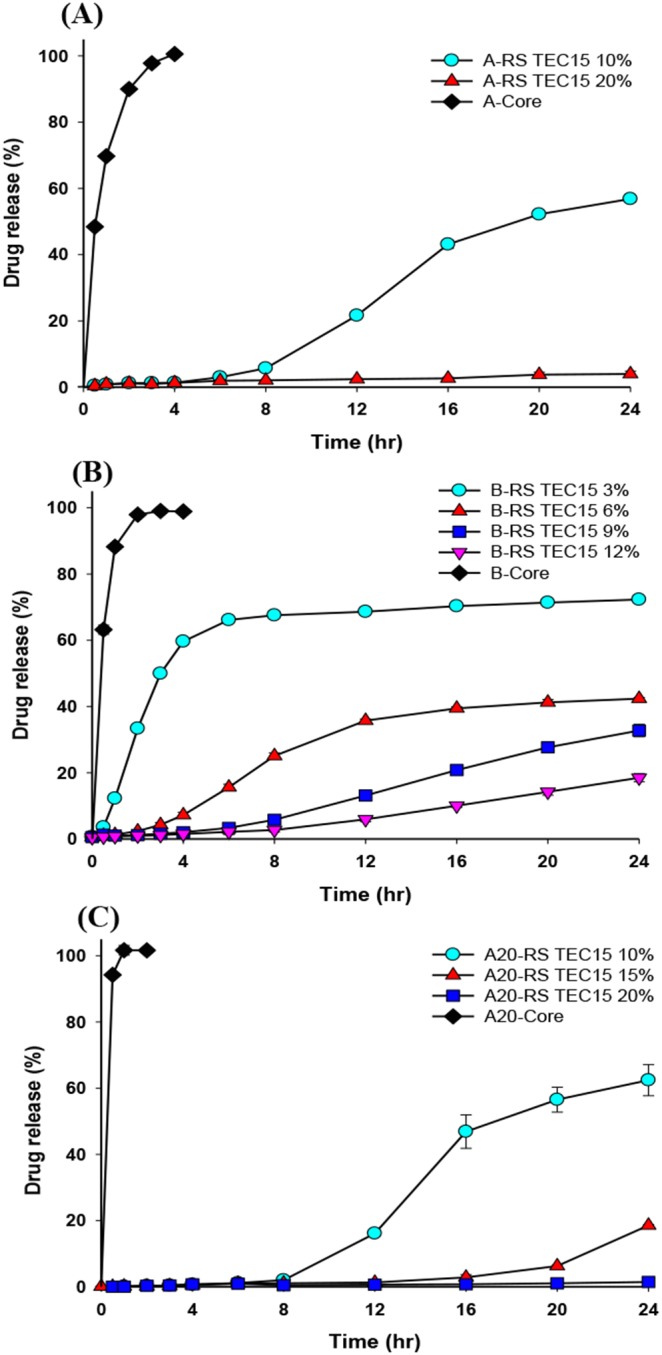
Losartan release profiles of formulations (A) A-RS TEC15 and A-Core, (B) B-RS TEC15 and B-Core, and (C) A20-RS TEC15 and A20-Core in pH 1.2 buffer.

**Figure 9:**
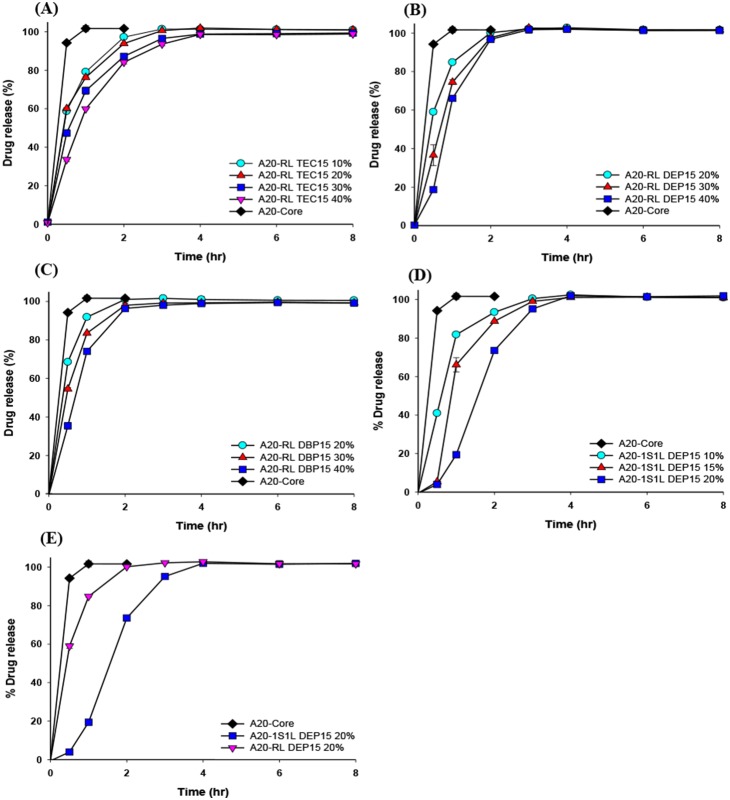
Losartan release profiles of A20-Core and formulations (A) A20-RL TEC15, (B) A20-RL DEP15, (C) A20-RL DBP15, and (D) A20–1S1L DEP15 in pH 1.2 buffer. (E) Effect of the coating membrane (Eudragit RL 30D: RS 30D at 1∶1 versus Eudragit RS 30D) on release profiles.

The drug-release profiles for those muFDDS formulations were characterized in simulated gastric fluid of pH 1.2 HCl dissolution medium using losartan as a model drug, and results are shown by **Figs. 8** and **9**. Results in **Fig. 8** show that when Eudragit RS was used as the coating film with the same plasticizer of TEC at 15%, a long sustained release profile of losartan with a coating level-dependent lag time was observed regardless of which core pellets (core A: **Fig. 8A**; core B; **Fig. 8B**; A20: **Fig. 8C**) were sprayed on. The lag time was obviously extended with an increasing coating level. This phenomenon was attributed to the lag time for membrane diffusion being proportional to the square of the thickness of the coating film (*t*_L_ = *h* ^2^/6*D*). Because of that, a complete release of 100% drug amount was not achievable within a 24-h period for any drug-containing pellets coated with Eudragit RS. On the contrary, faster release of losartan (complete release within 2?3 h) from A20 core pellets coated with Eudragit RL plasticized with TEC, DEP, and DBP at 15% was observed for all coating levels up to 40% (**Fig. 9A–C**). Apparently, a higher permeability of acidic medium across the Eudragit RL coating film than that of the Eudragit RS coating film accompanied by the freely soluble characteristic of losartan HCl in acidic medium was responsible for the difference in the release rates. Sungthongjeen et al. [15] reported that sustained release was able to completely release all drug within 11 h from pellets coated with Eudragit RL observed for theophylline, which has a lower solubility (sparely soluble) than losartan (freely soluble). Therefore, this muFDDS system is still potentially promising to deliver a sustained-release pattern for model drugs with lower solubilities or in a un-ionized form (acidic or basic form).

In consideration of the higher permeability of the Eudragit RL coating film (15% DEP as the plasticizer) having a shorter TPF and a longer period of floating and the lower permeability of Eudragit RS coating film having a greater extent of sustained release manner, a 1∶1 combination of Eudragit RS and RL was used as the coating film. **Figure 7E and 7F** demonstrate that an optimal TPF and a longer period of floating were achieved at a 10% coating level of Eudragit RS:RL at 1∶1 coating film. The drug release profile shown in **Fig. 9D** for a 10% coating level of Eudragit RS:RL at 1∶1 coating film was completed within 4 h, and the release rate also decreased with an increased coating level. At the same coating level of 20% as shown in **Fig. 9E**, the drug release from pellets coated with Eudragit RL: RS at 1∶1 was observed to be slower than that from pellets coated with Eudragit RL. This confirms that the combination of Eudragit RL and Eudragit RS had lower water permeability and was correspondingly expected to decrease the drug-release rate.

## Conclusions

Plasticized polymeric coating films for achieving rapid floating and a longer period of floating in a sustained-release manner were characterized for the effervescent muFDDSs developed in the present study. The water solubility of the plasticizer and its plasticized effect on the elongation of the polymeric coating film were the greatest influencing factors on the TPF and period of floating. The water solubility of the plasticizer in terms of water permeability is responsible for how soon the acidic medium can penetrate into the pellet core to generate CO_2_ gas by neutralizing the incorporated NaHCO_3_. The effect of the plasticizer on the polymeric coating film determines how easy it is for the resulting polymeric film to expand by the CO_2_ gas generated to a volume having a pellet density low enough for floating. The overall effects will determine the TPF. The water solubility also determines how long a pellet can float, since a plasticizer with optimal water solubility can be preserved in the coating film long enough to maintain its plasticized effect for the same expanded volume that has a density low enough for floating. It was concluded that DEP might be the plasticizer of choice among the plasticizers examined in this study for Eudragit RL to provide muFDDSs with a short TPF and a longer period of floating. However, the drug-release rate from muFDDSs using Eudragit RL plasticized with DEP was not sustainable by adjusting the coating level of the polymeric film. Alternatively, using a combination of Eudragit RL:RS of 1∶1 as the coating film prolonged drug release for a longer period. Drugs with a lower solubility or in an un-ionized form (acidic or basic form) would be another choice to prolong or sustain drug release rates from muFDDSs designed in this study.
